# Causes of acute respiratory failure in patients with small-vessel vasculitis admitted to intensive care units: a multicenter retrospective study

**DOI:** 10.1186/s13613-021-00946-x

**Published:** 2021-11-24

**Authors:** Aude Gibelin, Guillaume Dumas, Sandrine Valade, Marc Pineton de Chambrun, François Bagate, Mathilde Neuville, Francis Schneider, Loredana Baboi, Matthieu Groh, Jean-Herlé Raphalen, Jean-Daniel Chiche, Nicolas De Prost, Charles-Edouard Luyt, Claude Guérin, Eric Maury, Etienne de Montmollin, Alexandre Hertig, Antoine Parrot, Raphaël Clere-Jehl, Muriel Fartoukh

**Affiliations:** 1 Present Address: Service de Médecine Intensive Réanimation, Hôpital Tenon, Assistance Publique-Hôpitaux de Paris, Faculté de Médecine Sorbonne Université, 4 rue de la chine, 75020 Paris, France; 2 Service de Médecine Intensive Réanimation, Hôpital Saint Louis, Assistance Publique-Hôpitaux de Paris, Paris, France; 3 Service de Médecine Intensive Réanimation, Faculté de Médecine Sorbonne Université, Hôpital Saint Antoine, Assistance Publique-Hôpitaux de Paris, Paris, France; 4 Service de Médecine Intensive Réanimation, Hôpital Cochin, Assistance Publique-Hôpitaux de Paris, Paris, France; 5 Service de Médecine Intensive Réanimation, Faculté de Médecine Sorbonne Université, Hôpital Pitié Salpêtrière, Assistance Publique-Hôpitaux de Paris, Paris, France; 6 Service de Médecine Intensive Réanimation, Faculté de Santé de Créteil, Hôpitaux Universitaires Henri Mondor, Assistance Publique—Hôpitaux de Paris (AP-HP) and Groupe de Recherche Clinique CARMAS, Université Paris Est Créteil, Cedex 94010 Créteil, France; 7 Service de Médecine Intensive et Réanimation Infectieuse, Hôpital Bichat—Claude Bernard, Assistance Publique-Hôpitaux de Paris, Université Paris Diderot, IAME, UMR 1137, Paris, France; 8 Service de Médecine Intensive Réanimation, Hôpital Hautepierre, Strasbourg, France; 9 Service de Médecine Intensive-Réanimation Groupement Hospitalier Centre, Hôpital Edouard Herriot, Lyon, France; 10 Service de Médecine Interne, Hôpital Saint Louis, Assistance Publique-Hôpitaux de Paris, Paris, France; 11 Service de Réanimation Adultes, Hôpital Necker, Assistance Publique-Hôpitaux de Paris, Paris, France; 12 Service de Néphrologie, Hôpital Tenon, Assistance Publique-Hôpitaux de Paris, Faculté de Médecine Sorbonne Université, Paris, France

**Keywords:** Vasculitis, Diffuse alveolar hemorrhage, Intensive care, Acute respiratory failure

## Abstract

**Rationale:**

Acute respiratory failure (ARF) in patients admitted to the intensive care unit (ICU) with known or de novo small-vessel vasculitis (Svv) may be secondary to the underlying immune disease or to other causes. Early identification of the cause of ARF is essential to initiate the most appropriate treatment in a timely fashion.

**Methods:**

A retrospective multicenter study in 10 French ICUs from January 2007 to January 2018 to assess the clinical presentation, main causes and outcome of ARF associated with Svv, and to identify variables associated with non-immune etiology of ARF in patients with known Svv.

**Results:**

During the study period, 121 patients [62 (50–75) years; 62% male; median SAPSII and SOFA scores 39 (27–52) and 6 (4–8), respectively] were analyzed. An immune cause was identified in 67 (55%), and a non-immune cause in 54 (45%) patients. ARF was associated with several causes in 43% (*n*  = 52) of cases. The main immune cause was diffuse alveolar hemorrhage (DAH) (*n*  = 47, 39%), whereas the main non-immune cause was pulmonary infection (*n*  = 35, 29%). The crude 90-day and 1-year mortality were higher in patients with non-immune ARF, as compared with their counterparts (32% and 38% vs. 15% and 20%, respectively; both *p*  = 0.03), but was marginally significantly higher after adjusted analysis in a Cox model (*p*  = 0.053).

Among patients with a known Svv (*n*  = 70), immunosuppression [OR 9.41 (1.52–58.3); *p*  = 0.016], and a low vasculitis activity score [0.84 (0.77–0.93)] were independently associated with a non-immune cause, after adjustment for the time from disease onset to ARF, time from respiratory symptoms to ICU admission, and severe renal failure.

**Conclusions:**

An extensive diagnosis workup is mandatory in ARF revealing or complicating Svv. Non-immune causes are involved in 43% of cases, and their short and mid-term prognosis may be poorer than those of immune ARF. Readily identified predictive factors of a non-immune cause could help avoiding unnecessary immunosuppressive therapies.

**Supplementary Information:**

The online version contains supplementary material available at 10.1186/s13613-021-00946-x.

## Background

The International Chapel Hill Consensus Conference Nomenclature of Vasculitis aimed at characterizing systemic vasculitis as a function of the size of the vessels involved [1]. Accordingly, small-vessel vasculitis (Svv) are a group of diseases that include antineutrophil cytoplasmic antibody-associated vasculitis (AAV) and immune complex-associated small-vessel vasculitis. Retrospective series of patients with AAV admitted to the intensive care unit (ICU) indicate that acute respiratory failure (ARF) is the main reason for ICU admission [2–6], and that ARF is associated with a poor prognosis, with ICU mortality rates ranging from 11 to 52% [3–5, 7].

Diffuse alveolar hemorrhage (DAH) is the most common cause of ARF, identified in between 18 and 93% of cases [8, 9]. It is often associated with acute renal failure in the context of a pneumo-renal syndrome [10, 11]. Other respiratory disorders whether immune (i.e., pulmonary or bronchial granulomatosis, exacerbation of diffuse interstitial pneumonia, and others) or non-immune (e.g., cardiogenic pulmonary edema, bacterial or viral pneumonia, *Pneumocystis jirovecii* pneumonia) have also been identified as common causes of ARF in such patients [12–14]. Therefore, the recognition of the underlying cause as well as of its immune or non-immune mechanism is essential to initiate the most appropriate treatment of ARF in a timely fashion. To date, few studies have analyzed the causes of ARF in patients with systemic vasculitis admitted to the ICU. We carried out a retrospective multicenter study to assess clinical presentation, relative distribution of causes, and prognosis of ARF associated with Svv (either at the time of diagnosis of Svv or during its course), and to identify variables associated with a non-immune cause of ARF in patients with known Svv.

## Methods

### Study design

We conducted an 11-year multicenter retrospective non-interventional study in 10 French ICUs from January 2007 to January 2018. Eligible patients were identified from hospital records in each participating center by the local investigator, using the International Classification of Diseases, Ninth Revision (ICD-9) codes and the following keywords: “microscopic polyangiitis” (MPA), “granulomatosis with polyangiitis” (GPA, formerly Wegener’s granulomatosis), “eosinophilic granulomatosis with polyangiitis” (eosinophilic GPA, formerly Churg–Strauss syndrome), “anti-glomerular basement membrane disease” (GBM, or Goodpasture syndrome), with corresponding ICD-9 codes M31.7 (MPA), M31.3 (GPA), M30.1 (eosinophilic GPA), and N08.5X-005 or M31.0  +  (GBM). Some patients were previously included in the Connecticut registry that was also used for the 2009–2013 period [15]. All medical records of eligible patients were reviewed by the authors, and only patients with ARF (according to the following criteria: respiratory rate over 25 breaths/min or other signs of respiratory distress or a PaO_2_/FiO_2_ ratio  <  300 mmHg) were included. According to the French legislation (L.1121-1 paragraph 1 and R1121-2, Public Health Code), neither informed consent nor approval of an ethics committee is required for anonymous data extraction and analysis of patients’ medical files.

### Subjects, data collection and definitions

The patients included were 18-year old or older, admitted to the ICU for ARF associated with Svv either at the time of diagnosis of Svv or during its course. According to the Chapel Hill classification and/or American College of Rheumatology classification criteria [1], MPA, GPA, and eosinophilic GPA were considered, as well as anti-GBM antibody disease, as their clinical presentation and therapeutic management may be fairly similar, while other immune complex-associated Svv [cryoglobulinemic vasculitis, immunoglobulin A vasculitis, hypocomplementemic urticarial vasculitis (anti-C1q vasculitis)] were excluded from the analysis.

Acute respiratory failure was defined as a respiratory rate over 25 breaths/min or other signs of respiratory distress including active abdominal breathing, paradoxical breathing, impaired consciousness, or a PaO_2_/FiO_2_ ratio  <  300 mmHg.

When patients had several ICU admissions in the participating centers during the study period, only the first admission was considered for the analysis of clinical characteristics and outcome of immune compared to non-immune ARF, whereas the most recent admission was considered for the analysis of characteristics associated with non-immune cause of ARF in patients with known Svv.

For each patient, the following variables were recorded, using a standardized and anonymized case report form: demographics (age, gender), severity scores on ICU admission (Sequential Organ Failure Assessment and Simplified Acute Physiology Score II) [16, 17], vasculitis activity and prognosis scores using the Birmingham Vasculitis Activity Score (BVAS) [18] and the 2011 revised Five-Factor Score (FFS) [19]. The BVAS is a 1-page form comprising 34 predefined items grouped into 9 separate organ systems, and measuring disease activity. The Five-Factor Score (FFS) is a prognostic score calculated upon admission that includes age, serum creatinine level (> 150 μmol/l or  < 150 μmol/l), presence of severe gastrointestinal tract involvement, cardiomyopathy, and ear, nose, and throat involvement. The revised FFS was used to assess vasculitis activity; the Acute Kidney Injury (AKI) upon ICU admission was defined by the need for renal replacement therapy according to KDIGO score [20]. The main comorbidities were collected in the history data of the hospital report. Severe chronic renal failure was defined as a creatinine clearance of 30 ml/min or less, or chronic dialysis. Time interval between the first respiratory signs and ICU admission, clinical (including respiratory and extra-respiratory manifestations) and laboratory findings on admission, chest X-ray and chest CT scan on admission, and cytological and microbiological analyses of broncho-alveolar lavage (BAL) fluid if performed, were also recorded. An hemorrhagic BAL fluid was defined as a macroscopic bloody or pinky fluid with cytologic analysis impossible (too many red blood cells or coagulation). The definition of DAH was based on the following criteria: the clinical and radiological presentation was compatible (hemoptysis, new pulmonary infiltrates and anemia) and the BAL fluid was hemorrhagic [21].

Therapeutic interventions recorded included the need for vital organ support during ICU stay (mechanical ventilation, extra-corporeal membrane oxygenation (ECMO), renal replacement therapy, vasopressors), and the administration of immunosuppressive treatments (steroids, cyclophosphamide, rituximab, intravenous immunoglobulins or plasma exchange, others). Lengths of stay in the ICU, ICU and hospital mortality rates, duration of follow-up after ICU discharge, as well as 28-day, 90-day and 1-year mortality rates were recorded.

### Classification of the cause(s) of acute respiratory failure

**Fig. 1:**
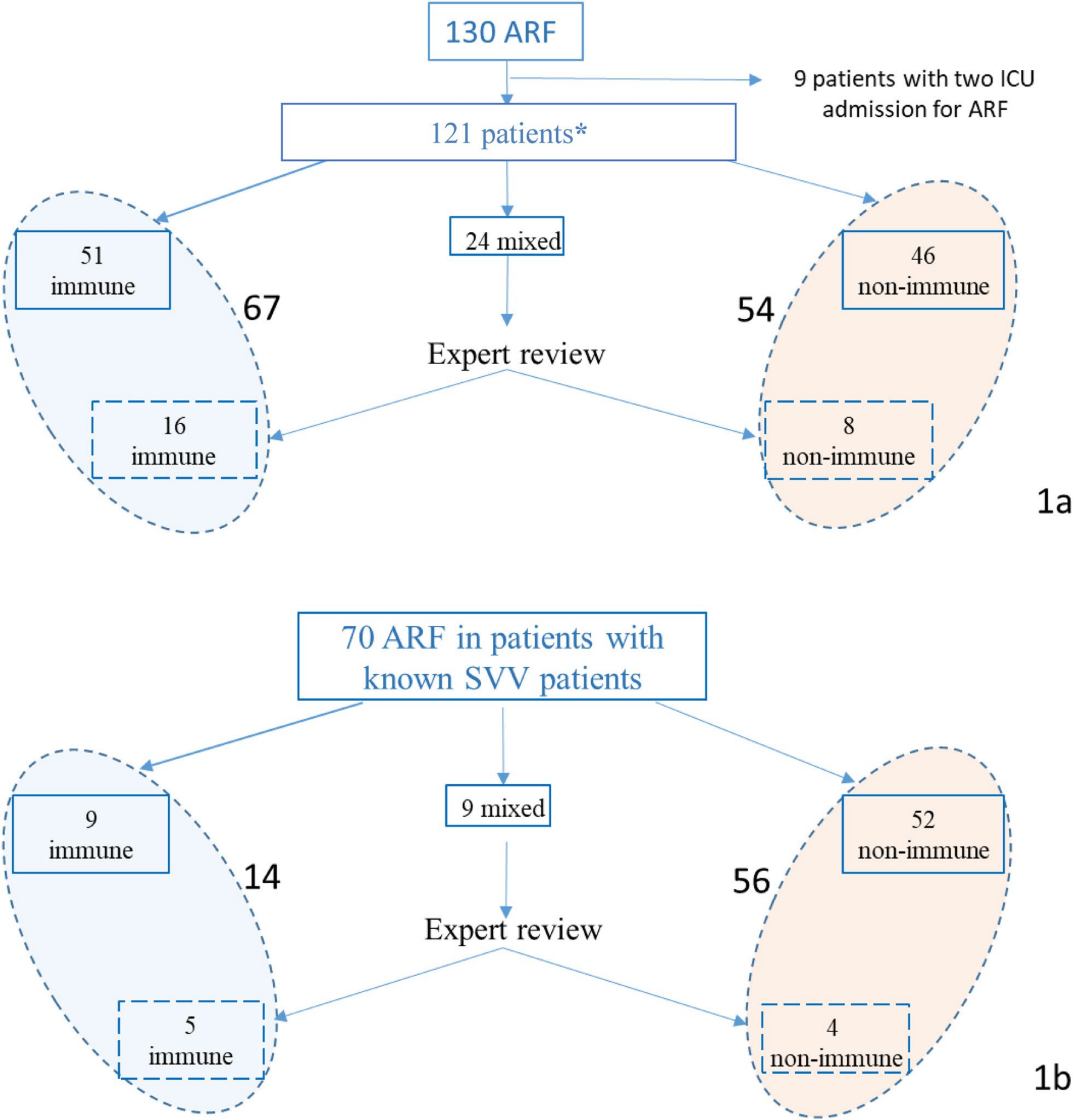
Flowchart. Classification of the cause(s) of acute respiratory failure (ARF). **a** Analysis of causes of ARF and of survival (*n*  =  121). *Only the first admission was considered. **b** Analysis of risk factors for non-immune ARF in patients with known Svv (*n*  = 70). Only the most recent episode was considered in the analysis of risk factors for non-immune ARF in patients with known Svv

All identified causes of ARF were collected for the descriptive analysis. The primary cause of ARF was recorded as that diagnosed by the clinician in charge of the patient (corresponding to the conclusion of hospital record) and reviewed by the principal investigator (AG) according to history, clinical, laboratory and radiological data. Then, patients were categorized as having “immune ARF” when having respiratory failure secondary to the Svv exacerbation, according to the Chapel Hill classification and/or American College Rheumatology classification criteria (i.e., diffuse alveolar hemorrhage, pulmonary or tracheal/bronchial granulomatosis, interstitial lung disease, asthma, or myocarditis) [1]. Patients were categorized as having “non-immune ARF” if other etiologies or mechanisms were identified. In case of discrepancy or of a combination of immune and non-immune causes, adjudication of the predominant cause(s) of ARF was performed by two other experts (AP, AH). Three etiological groups of ARF were thus defined: immune, non-immune, and mixed ARF (Additional file 1: Table S1). Twenty-five cases were reviewed by the two experts essentially because of a combination of immune and non-immune causes (24/25 cases), and one because of a discrepancy between recorded diagnosis and the investigator. For the analyses, due to similar clinical presentation and therapeutic approaches, patients with mixed ARF and a predominant immune cause or a predominant non-immune cause were grouped with those having immune ARF or non-immune ARF, respectively. Thus, only two groups were considered in these analyses: “immune ARF” and “non-immune ARF” (Fig. 1).

### Data presentation and statistical analysis

We first aimed to describe and compare the clinical presentation, relative distribution of causes, and prognosis of immune and non-immune ARF groups. We then focused on the subset of patients with a known Svv on ICU admission to examine clinical variables associated with a non-immune cause of respiratory failure (Fig. 1).

Continuous variables are reported as median (interquartile range IQR 25–75) and categorical variables as number and percentages. The Chi-square or the Fisher exact tests, when appropriate, were used to compare qualitative variables, and the Wilcoxon rank-sum test was used to compare quantitative variables. All tests were two-sided, with *p*  < 0.05 indicating statistical significance.

The effect of the immune or non-immune etiology of ARF on 1-year survival was estimated with a Cox proportional hazards model, with adjustment for four baseline covariates: the SAPS2 score (as a dichotomous variable,  < / > 40), the duration (months) of Svv follow-up until ARF, immunosuppression, and severe chronic kidney disease. Results are reported as hazard ratio (HR) with 95% confidence interval and as Kaplan–Meier curves. The same analyses have been performed using only patients with pure immune or non-immune etiology of ARF (exclusion of mixed ARFs) to eliminate a possible misclassification bias.

In the subset of patients with a known Svv on ICU admission, univariable analysis first assessed the association between each variable and the cause of ARF. All confidence intervals were calculated at the 95% level. Variables selected by univariable analysis (*p*  < 0.1) were entered in a logistic regression model to identify the predictors of a non-immune cause of ARF, using a stepwise backward logistic regression. Statistical tests were performed by using Stata™ 15.1 software (StataCorp, College Station, Texas, USA).

## Results

### Study population

Between January 2007 and January 2018, 121 patients were hospitalized in the participating ICUs for ARF, of whom 9 had two ICU admissions. In this subgroup, only the first episode was considered in the analysis of causes of ARF and of survival, whereas only the most recent episode was considered in the analysis of risk factors for non-immune ARF in patients with known Svv. Thus, there were 67 and 54 patients with immune and non-immune ARF included in the survival analysis, whereas these numbers were 14 and 56, respectively, in the risk factors analysis (Fig. 1).

### Causes of acute respiratory failure

Patients were admitted in the participating ICUs for a first episode of ARF either at Svv disease onset (*n*  = 55, 45%) or later during follow-up (*n*  = 66, 55%). Among them, 52 (43%) had GPA, 37 (31%) had MPA, 19 (16%) had EGPA, 11 (9%) had anti-GBM antibody disease, and 2 (2%) had undifferentiated ANCA vasculitis.

According to the cause(s) and mechanism(s) of ARF identified, immune and non-immune causes were evenly distributed among the 121 patients, with 67 (55%) having an immune cause, and 54 (45%) a non-immune cause. DAH predominated among immune causes of ARF (*n*  = 47; 70%), followed by exacerbation of interstitial lung disease, and pulmonary or bronchial granulomatosis, whereas non-immune causes were mainly related to lower respiratory tract infections (confirmed or presumed, *n*  = 35; 65%) or cardiac (acute pulmonary edema, *n*  = 18; 33%) etiologies (Table 1).

**Table 1:** Causes of acute respiratory failure associated with Svv (*n*  =  121)

Cause of acute respiratory failure (ARF)	*n*	(%)
**Immune ARF**	67	(55)
Diffuse alveolar hemorrhage (DAH)	47	(70)
DAH with pulmonary renal syndrome	38	(57)
Interstitial lung disease	11	(16)
Pulmonary or tracheal/bronchial granulomatosis	9	(13)
Asthma	8	(12)
Myocarditis	4	(6)
Non-immune cause associated with immune ARF^a^	16	(24)
**Non-immune ARF**	54	(45)
Pulmonary infection (confirmed or suspected)	35	(65)
Confirmed^d^	24	(44)
Bacterial^d^	15	(28)
Viral	5	(9)
*Pneumocystis* *jirovecii*	5	(9)
Suspected	11	(20)
Acute pulmonary edema	18	(33)
Systolic or diastolic dysfunction of the left ventricle	12	(22)
Fluid overload (no cardiac dysfunction)	6	(11)
Pulmonary embolism	6	(11)
Pneumothorax (spontaneous or iatrogenic)	4	(7)
Tumoral^c^	3	(6)
Immune cause associated with non-immune ARF^b^	8	(15)

Only the first admission was considered for the description of the causes of immune and non-immune ARF

Several causes were diagnosed in 52 (43%) patients: 14 patients with two or more non-immune causes, 14 patients with two or more immune causes, and 24 with a combination of immune and non-immune causes

^a^Non-immune causes were associated with immune ARF in 16 patients, including acute pulmonary edema (*n*  = 9), pulmonary infection with microbiological documentation (*n*  = 6), and pulmonary embolism (*n*  = 2)

^b^Immune causes were associated with non-immune ARF in 8 patients, including alveolar hemorrhage (*n*  = 3), pulmonary/bronchial granulomatosis (*n*  = 2), interstitial lung disease (*n*  = 2), and 1 upper airways obstruction

^c^Lung cancer (*n*  = 2) and leukemia (*n*  = 1)

^d^Two patients had a fungal infection associated with a bacterial infection: one bronchopulmonary aspergillosis and one invasive pulmonary aspergillosis, and two patients had both bacterial and viral infection

At least two causes were recorded in 30 (45%) and 22 (41%) patients, respectively, in the immune and non-immune ARF group, including 16 and 8 patients, respectively, having an immune cause associated with a non-immune cause (Table 1).

### Patients characteristics

The studied patients (62% males) had a median age of 62 (50–75) years. Those with non-immune ARF were older, had more often comorbid conditions, and had a diagnosis of vasculitis since a median of four years, whereas 76% of those with immune ARF had the diagnosis established during the ICU stay. Fourteen patients had a previous ICU stay, including 9 for a previous episode of ARF. The median SAPSII and SOFA scores were, respectively, 39 (27–52) and 6 (4–8) on ICU admission, and although SAPSII was higher because of a higher age in the non-immune ARF group, the organ failure score SOFA did not differ between the two groups. The time between the first respiratory signs and ICU admission was shorter in the non-immune ARF group, as compared with the immune ARF group, with 54% of patients with non-immune ARF having symptoms progressing for less than 3 days vs. 24% of those with immune ARF (*p*  = 0.001). Most patients (86%) had general symptoms (asthenia, fever, weight loss). Extra-respiratory signs [cutaneous (rash, purpura), rheumatologic (arthralgia, arthritis, Raynaud’s syndrome), muscular (myalgia), neurological (mono- or multi-neuritis, focal deficit), gastrointestinal (abdominal pain, gastrointestinal bleeding), ENT (dysphonia, nasal crust), lymphadenopathy, ophthalmologic (scleritis), abnormalities of urinary sediment (proteinuria, hematuria)] were more frequent in patients with immune ARF, as compared with their counterparts (Table 2).

**Table 2:** Characteristics of 121 patients with ARF associated with Svv on ICU admission

	All patients *n* = 121		Immune ARF *n* = 67		Non-immune ARF *n* = 54		*p*
Demographics							
Age, year	62	[50–75]	59	[44–72]	68	[57–76]	0.008
Male gender, n (%)	75	(62)	38	(57)	37	(69)	0.184
Diabetes	34	(28)	16	(24)	18	(33)	0.250
Cardiovascular disease^a^, *n* (%)	63	(52)	27	(40)	36	(67)	0.004
Respiratory disease^b^, *n* (%)	36	(30)	14	(21)	22	(41)	0.002
Immunosuppression^c^, *n* (%)	55	(45)	10	(15)	45	(83)	< 0.0001
Severe chronic renal failure^d^, *n* (%)	23	(19)	4	(6)	19	(35)	< 0.0001
Small vessel vasculitis [Svv]							
Etiology of Svv							0.358
GPA	52	(43)	28	(42)	24	(44)	
MPA	37	(31)	24	(36)	13	(24)	
EGPA	19	(16)	9	(13)	10	(19)	
GBM	11	(9)	6	(9)	5	(9)	
Unspecified ANCA vasculitis	2	(2)	0	(0)	2	(4)	
Time from Svv diagnosis to ICU admission, months	1.5	[0–58]	0	[0–0]	48	[4–132]	< 0.0001
Svv diagnosis in ICU, *n* (%)	55	(45)	51	(76)	4	(7)	< 0.0001
BVAS (Birmingham Vasculitis Activity Score)	15	[1–21]	21	[15–25]	0	[0–9]	< 0.0001
Revised FFS (Five-factor score)	1	[0–2]	1	[1, 2]	0	[0–1]	0.0004
Clinical presentation upon ICU admission							
Moderate-to-severe ARDS	41	(34)	30	(45)	11	(20)	0.005
Arterial hypertension	25	(21)	14	(21)	11	(20)	0.943
Shock	20	(17)	7	(10)	13	(24)	0.045
Neurological (GCS ≤ 13)	19	(16)	8	(12)	11	(20)	0.205
Time from respiratory symptoms to ICU admission, days, *n* (%)	3	[2–4]	4	[3, 4]	2	[1–3]	0.0002
< 3 days	45	(38)	16	(24)	29	(54)	0.001
≥ 3 days	76	(63)	51	(76)	25	(46)	
Extra-respiratory symptoms^e^, *n* (%)	104	(86)	61	(91)	43	(80)	0.072
Specific extra-respiratory symptoms^f^, *n* (%)	63	(52)	47	(70)	16	(30)	< 0.0001
Laboratory features upon ICU admission							
Hemoglobin, g/dl	9.9	[8–12]	8.8	[7.2–10.8]	11	[8.8–12.4]	0.0012
Leucocytes, giga/l	13.0	[8.2–16.5]	13.9	[9.2–18.2]	10.7	[7.2–15.6]	0.020
Plasma creatinine level, µmol/l	200	[88–398]	229	[79–422]	188	[96–300]	0.75
Hematuria [> 10^4/ ml, *n* (%)^g^	54	(45)	44	(66)	10	(19)	< 0.0001
Positive ANCA and/or GBM^g^	75	(62)	57	(85)	18	(33)	< 0.0001
Severity criteria upon ICU admission							
SAPS II	39	[27–52]	37	[24–49]	42	[30–54]	0.047
SOFA	6	[4–8]	6	[3–8]	5.5	[4–8]	0.667
Vital support administered during the first 48 h, *n* (%)							
Mechanical ventilation	78	(64)	45	(67)	33	(61)	0.49
Vasopressors	34	(28)	18	(27)	16	(30)	0.113
Renal replacement therapy	48	(40)	28	(42)	20	(37)	0.595

Only the first admission was considered for the analysis of the characteristics of immune compared to non-immune ARF. Continuous variables are reported as median [interquartile range (IQR) 25–75]. Categorical variables are reported as number (percentages)

*Svv* small vessel vasculitis; *BVAS* Birmingham Vasculitis Activity Score; *FFS* Five-Factor Score; *GPA* granulomatosis with polyangiitis; *MPA* microscopic polyarteritis; *EGPA* eosinophilic GPA; *GBM* anti-GBM antibodies disease; *ANCA* anti-neutrophil cytoplasmic antibodies; *GSC* Glasgow Coma Scale; *SAPSII* and *SOFA* Simplified Acute Physiology Score II and Sequential Organ Failure Assessment score

^a^Arterial hypertension, cardiac failure and/or ischemic heart disease

^b^COPD or asthma or interstitial lung disease

^c^Active cancer, HIV or immunosuppressive treatment

^d^Glomerular filtration rate  < 30 ml·min-1 over 1 month or more

^e^Fever or asthenia or weight loss

^f^Cutaneous (skin rash, purpura), rheumatic (arthralgia, arthritis, Raynaud’s syndrome, myalgia), neurological, gastrointestinal (abdominal pain, gastrointestinal bleeding), ENT (dysphonia, nasal crusts), lymphadenopathy

^g^Data missing, respectively, for 11 patients and 13 patients (presence of hematuria and auto antibodies)

### ICU investigations, management and outcomes

Diagnostic workup during the first 48 h of ICU admission, included chest CT scan (*n*  = 95, 79%), fiberoptic bronchoscopy with BAL (*n*  = 69; 57%), trans-thoracic echocardiography (*n*  = 109; 90%), and autoimmunity tests (*n*  = 108; 89%). Chest CT scan findings included mostly ground-glass attenuation and alveolar consolidation, and were more common in the immune ARF group than in the non-immune ARF group (88% and 79% vs. 49% and 44%, *p*  < 0.0001). An hemorrhagic BAL fluid was found in 38 (62%) of the BAL performed, more often in the immune than in the non-immune ARF group (33/47 vs. 5/22; *p*  < 0.0001) (Additional file 1: Table S2).

Invasive (*n*  = 60) or non-invasive (*n*  = 30) mechanical ventilation was required altogether in 78 patients (64%), mainly for ARDS. Ventilator-associated pneumonia (VAP) developed more frequently in the immune ARF group, as compared with the non-immune ARF group (*p*  = 0.009). Overall ICU and hospital mortality rates were 19% and 24%, respectively. Patients with non-immune ARF had higher crude mortality rate in the hospital, at 90 days and at 1 year than their counterparts (Table 3). Most patients with immune ARF received immunosuppressive drugs, as compared with their counterparts (97% vs. 22%; *p*  < 0.001), with corticosteroids, often in high-dose pulses, as the most frequent drug administered. Other treatments administered are reported in Table 3. In the non-immune ARF group, immunosuppressive treatments, mainly steroids, were administered for bronchospasm (*n*  = 3), chemotherapy (*n*  = 2) or drug-induced lung injury (*n*  = 1).

**Table 3:** Management during the ICU stay and outcomes of 121 patients with ARF associated with Svv

	All patients *n* = 121		Immune ARF *n* = 67		Non-immune ARF *n* = 54		*p*
Immunosuppressive therapy, *n* (%)	77	(64)	65	(97)	12	(22)	< 0.0001
Systemic steroids	75	(62)	65	(97)	10	(19)	< 0.0001
High-dose pulses	65	(54)	60	(90)	5	(9)	< 0.0001
Cyclophosphamide	45	(37)	42	(63)	3	(6)	< 0.0001
Rituximab	14	(12)	13	(19)	1	(2)	0.003
Plasma exchange	37	(31)	34	(51)	3	(6)	< 0.0001
Other treatments during the first 48 h, *n* (%)							
Blood transfusion	51	(42)	40	(60)	11	(20)	< 0.0001
Antibiotics	106	(88)	60	(90)	46	(85)	0.47
Diuretics	42	(35)	22	(33)	20	(37)	0.63
Management and outcomes in the ICU							
Invasive mechanical ventilation, *n* (%)	60	(50)	34	(49)	26	(50)	0.94
ARDS, *n* (%)	62	(51)	40	(60)	22	(41)	0.038
Ventilator-associated pneumonia, *n* (%)	24	(20)	19	(28)	5	(9)	0.009
Ventilator-free days at day 28, days	23	[5–28]	20	[5–28]	24	[7–28]	0.40
Shock (vasopressor treatment > 48 h)	37	(31)	21	(31)	16	(30)	0.84
Renal replacement therapy	57	(47)	35	(52)	22	(41)	0.21
Length of ICU stay (all patients)	8	[4–38]	11	[5–20]	5.5	[3–12]	0.014
Length of ICU stay of survivors only	10.5	[5–23]	13	[7–25]	7	[4–17]	0.018
Mortality, *n* (%)							
ICU	23	(19)	10	(15)	13	(24)	0.20
Hospital	29	(24)	11	(16)	18	(33)	0.030
Day 90^a^	27/118	(23)	10/65	(15)	17/53	(32)	0.032
1 year^a^	33/118	(28)	13/65	(20)	20/53	(38)	0.033

Continuous variables are reported as median [interquartile range (IQR) 25–75]. Categorical variables are reported as number (percentage)

^a^Three patients lost to follow-up before d90 (1 in non-immune and 2 in the immune ARF group)

**Fig. 2:**
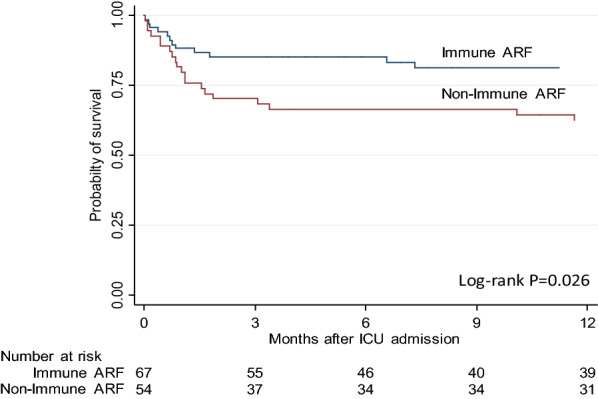
Kaplan–Meier estimates of the survival rates at 1 year from ICU admission for acute respiratory failure (ARF) in patients with Svv, comparing patients with a predominant immune (red curve) or non-immune (black curve) cause of ARF. Only the first admission was considered for the survival analysis

Patients were followed for a median of 18 [2–44] months after ICU admission. The Kaplan–Meier graph showed a lower probability of one-year survival after ICU admission in the non-immune ARF group, as compared with the immune ARF group (Fig. 2; *p*  = 0.026, log-rank test). The 90-day survival was also higher in the immune ARF group (Table 3). After adjustment for time since onset of disease, immunosuppression, severe renal failure and the severity of acute illness score SAPS II (which includes age) in a Cox model, non-immune ARF was marginally associated with a poorer 1-year survival (*p*  = 0.052, Additional file 1: Table S3). In the Cox model for survival included only the patients with pure etiology of ARF, the non-immune ARF was also associated with a poorer 1-year survival (*p*  = 0.034). However, when adjusted for the SAPSII score, the duration of Svv follow-up until ARF, immunosuppression, and severe chronic kidney disease, only the SAPSII score remained independently associated with survival (*p*  = 0.009), while the non-immune etiology was marginally associated with survival (*p*  = 0.098) (Additional file 1: Table S3).

### Patients with ARF complicating a known Svv

Of the 121 patients, 70 suffered ARF complicating a known Svv, including 14 and 56 with a predominant immune or non-immune cause, respectively, when considering the second episode only in the 9 patients having two ICU admissions for ARF. Except for immunosuppression, the two groups were well balanced regarding age and other underlying diseases (Table 4). The predictive factors of a non-immune cause were immunosuppression [OR 9.41 (1.52–58.3); *p*  = 0.016] and a low vasculitis activity score [OR 0.78 (0.69–0.89); *p*  < 0.0001] (per point of the BVAS), after adjustment on severe renal failure, time since onset of Svv and time from respiratory symptoms to ICU admission (Table 5).

**Table 4:** Characteristics of ARF complicating a known Svv on ICU admission (n = 70)

	All patients *n* = 70		Immune ARF *n* = 14		Non-immune ARF *n* = 56		*p*
Demographics							
Age, year	67	[57–76]	67.5	[57–77]	67.0	[57–76]	0.498
Male gender, *n* (%)	47	(67)	8	(57)	39	(70)	0.373
Cardiovascular disease^a^, *n* (%)	45	(64)	7	(50)	38	(68)	0.212
Diabetes	24	(34)	5	(36)	19	(34)	0.900
Respiratory disease^b^, *n* (%)	27	(39)	3	(21)	24	(43)	0.156^g^
Immunosuppression^c^, *n* (%)	57	(81)	7	(50)	50	(89)	0.001
Severe renal dysfunction^d^, *n* (%)	28	(40)	3	(21)	25	(45)	0.138^g^
Long-term dialysis, *n* (%)	17	(24)	1	(7)	16	(29)	0.162^g^
Small vessels vasculitis [Svv]							
Time from Svv diagnosis to ICU admission, months	48	[8–124]	21	[8–60]	56	[9.5–132]	0.077
BVAS (Birmingham Vasculitis Activity Score)	2	[0–9]	17.5	[8–21]	0	[0–6]	< 0.00001
Revised FFS (Five-factor score)	0	[0–1]	2	[1, 2]	0	[0–1]	< 0.0001
Clinical presentation							
Moderate-to-severe ARDS	17	(24)	5	(36)	12	(21)	0.304^g^
Arterial hypertension	15	(21)	4	(29)	11	(20)	0.480^g^
Shock	16	(23)	1	(7)	15	(27)	0.164^g^
Neurological	16	(23)	1	(7)	15	(27)	0.164^g^
Time from respiratory symptoms to ICU admission, med days	2.5	[1–3]	3.5	[2–4]	2	[1–3]	0.0367
< 3 days, *n* (%)	35	(50)	4	(29)	31	(55)	0.073^g^
≥ 3 days	35	(50)	10	(71)	25	(45)	
Extra respiratory symptoms^e^, *n* (%)	54	(77)	10	(71)	44	(79)	0.569
Specific extra respiratory symptoms^f^, *n* (%)	20	(29)	6	(43)	14	(25)	0.186
Laboratory features							
Hemoglobin, g/l	10.6	[9–12.3]	9.8	[7.8–11.5]	11	[9.3–12.4]	0.139
Plasma creatinine level, µmol/l	197	[95–327]	178	[73–379]	197	[97–299]	0.572
Hematuria [> 10^4/ml], *n* (%)^h^	17	(24)	7	(50)	10	(18)	0.057^g^
Proteinuria g/l [> 0, 3], *n* (%)^h^	23	(33)	4	(29)	19	(34)	0.186^g^
Presence of auto antibodies^h^	25	(36)	11	(79)	14	(25)	0.001
Severity criteria at ICU admission							
SAPS II	45.5	[29–57]	35	[26–54]	46	[30–59]	0.319
SOFA	5	[4–9]	4.5	[3–8]	6	[4–9]	0.181
Organ support administered during the first 48 h, *n* (%)	64	(79)	22	(85)	42	(76)	0.395
Mechanical ventilation	48	(69)	10	(71)	38	(68)	0.797
Vasopressors	22	(31)	3	(21)	19	(34)	0.368
Renal replacement therapy	25	(36)	6	(43)	19	(34)	0.533

Continuous variables are reported as median [interquartile range (IQR) 25–75]. Categorical variables are reported as number (percentages)

*Svv* small vessel vasculitis; *BVAS* Birmingham Vasculitis Activity Score; *FFS* Five Factor Score; *ANCA*, *SAPSII* Sequential Organ Failure Assessment and Simplified Acute Physiology Score II; *GPA* granulomatosis with polyangiitis; *MPA* microscopic polyarteritis; *EGPA* eosinophilic GPA; *GBM* anti GBM anti bodies disease; *SOFA* Sepsis-related Organ Failure Assessment; *GSC* Glasgow coma scale

^a^Heart failure or arterial hypertension or coronary disease

^b^COPD or asthma or interstitial lung disease

^c^Active cancer, HIV or immunosuppressive treatment

^d^Glomerular filtration rate  < 30 ml·min-1 over 1 month

^e^Fever or asthenia or weight loss

^f^Cutaneous (skin rash, purpura), rheumatic (arthralgia, arthritis, Raynaud’s syndrome, myalgia), neurological, gastrointestinal (abdominal pain, gastrointestinal bleeding), ENT (dysphonia, nasal crusts), lymphadenopathy

^g^Fisher’s exact test

^h^Data missing, respectively, for 10, 21 and 13 patients (presence of hematuria, proteinuria and auto antibodies)

**Table 5:** Univariable and multivariate analyses of factors associated with a non-immune cause in the subgroup of patients with ARF complicating a known Svv (n = 70)^a^

	Immune ARF (*n* = 14)	Non-immune ARF (*n* = 56)	Univariable analysis	*p*	Multivariate analysis	*p*
			OR [95% CI]		OR [95%CI]	
Immunosuppression	7 (50)	50 (89)	8.33 [2.17–32.05]	0.002	9.41 [1.52–58.3]	0.016
Severe chronic renal failure, *n* (%)	3 (21)	25 (45)	2.95 [0.74–11.76]	0.124	NR	0.33
Time from Svv diagnosis to ICU admission, months	21 [8–60]	56 [9.5–132]	1.009 [0.999–1.020]^b^	0.084	NR	0.099
BVAS (Birmingham Vasculitis Activity Score)	17.5 [8–21]	0 [0–6]	0.85 [0.78–0.92]^b^	< 0.0001	0.84 [0.77–0.93]	< 0.0001
Time from resp. symptoms to ICU admission, < 3 days	4 (29)	31 (55)	3.10 [0.87–11.07]	0.082	NR	0.13

*NR* not retained in the model at the *p*  =  0.05 significance level

^a^Including the second admission only in the 9 patients with 2 ICU admissions

^b^Per unit (per month since the diagnosis of ARF or per BVAS point)

## Discussion

This study is the first multicentric study to focus specifically on ARF associated with Svv in critically ill patients. The main findings are as follows: (1) DAH accounts for one-third of the causes of ARF; (2) the prognosis of non-immune ARF may be poorer than that of immune ARF; and (3) among patients with ARF complicating a known Svv, immunosuppression and a low BVAS are associated with a non-immune cause of ARF.

The causes of ARF were separated into two groups: non-immune (45%), and immune (55%). Of note, ARF was related to several causes in over one-third of cases, including about 20% of ARF episodes where ARF is caused both by an immune and a non-immune cause. In these mixed episodes, a predominant immune or non-immune cause was identified after expert review.

The main immune cause was DAH. The prevalence of DAH was lower than that usually described [3, 4], accounting for one-third of the causes of ARF, overall (*n*  = 47; 39%). This discrepancy may be explained by several factors: (i) some series of ARF associated with Svv have focused on vasculitis exacerbations exclusively [3, 4]; (ii) it is conceivable that some unusual causes of immune ARF, such as interstitial lung disease (ILD) or pulmonary or bronchial granulomatosis, may have not been included in previously published series [22]. The second cause of immune ARF was related to ILD, that may present as an usual interstitial pneumonia particularly when associated with MPA [12], or as an acute or subacute eosinophilic pneumonitis associated with EGPA [23]. Pulmonary or bronchial granulomatosis, rarely described in the intensive care unit [13, 24], represented the third cause of immune ARF in our series. Other immune causes were severe acute asthma and myocarditis, two less frequent conditions, but usually associated with poor outcome [25]. The well-known association between the type of vasculitis and various respiratory disorders was confirmed in our series of critically ill patients, with DAH being more likely associated with GBM and MPA [8, 9, 26], granulomatosis with GPA [13, 24] and eosinophilic pneumonitis and asthma with EGPA [23] (Additional file 1: Table S4).

The main non-immune causes of ARF were mainly infectious (clinically suspected or confirmed lower respiratory tract infection) and cardiac. Pulmonary embolism was rarely the main cause of ARF, but was an associated cause in 6% of cases. This higher prevalence of pulmonary embolism than that observed in the general population is consistent with the literature [27].

It is noteworthy that ARF was secondary to several causes in 42% of cases. Thus, a complete etiological work-up including chest CT scan, fiberoptic bronchoscopy with BAL, trans-thoracic echocardiography and autoimmunity tests, should be considered in all patients. At the individual level, this etiological investigation is of major importance given the number of possible causes of ARF, and the presence of several causes in nearly half of the cases. In our series, the clinical presentation of immune ARF differed from that of non-immune ARF in several ways. A longer time elapsed between the first respiratory symptoms and ICU admission in patients with immune ARF, as compared with their counterparts. The patients with immune ARF had less comorbidities (immunosuppression, chronic renal, cardiovascular, or respiratory disease) and a higher BVAS score, and more specific extra-respiratory clinical signs. Additional investigations showed higher rates of chest-CT ground glass attenuation, hemorrhagic BAL, and presence of antibodies.

Altogether, the overall ICU and hospital mortality rates averaged 25%, and were comparable to those reported in the literature [3, 7]. However, the probabilities of 90-day and one-year survivals after ICU admission tended to be lower in the non-immune ARF group, as compared with the immune ARF group. In our series, immunosuppression and a low BVAS were independently associated with a non-immune cause of ARF in patients with known Svv. In this subset of patients, the early identification of a non-immune cause may help to avoid unnecessary immunosuppressive therapies, and our study highlights the worse prognosis and lower probability of long-term survival in patients with ARF of non-immune causes.

## Limitations

Our study has several methodological limitations: 1/ given its retrospective design, a number of information were missing, notably regarding imaging and BAL fluid analyses; 2/ variables associated with a non-immune ARF cause would need validation on an external cohort, which is challenging given the rarity of the diseases; 3/ finally, the small numbers of each of the causes within the etiological groups limited the power of the analyses. Last, medical practices may have changed over time and contributed to modify the outcomes, thus questioning the generalizability of our findings [28–30].

## Conclusion

We report a large cohort of ARF associated with Svv, among which DAH represented one-third of causes. ARF causes may be classified into two relevant groups—immune and non-immune—that differ by their clinical presentation and other characteristics, therapeutic management and prognosis. The outcome of patients having ARF of immune cause appears to be better than those having a non-immune cause. An extensive etiological diagnosis workup is therefore mandatory for these patients to avoid unnecessary immunosuppressive therapy, especially since the presence of several causes are common.

## Supplementary Information

**Additional file 1: Table S1.** Demographics and clinical manifestations of ARF upon ICU admission in 121 patients with Svv. **Table S2.** Diagnostic workup upon ICU admission of 121 patients with ARF associated with Svv. **Table S3.** One-year survival. **Table S4.** Causes of acute respiratory failure according to the etiology of Svv (n=121).

## Data Availability

All data generated or analyzed during this study are included in this published article (and its Additional files).
