# Supplementary material for: Causes of acute respiratory failure in patients with small-vessel vasculitis admitted to intensive care units: a multicenter retrospective study
Source: Ann Intensive Care. 2021 Nov 24;11:158. doi: 10.1186/s13613-021-00946-x (PMC8613321; doi:10.1186/s13613-021-00946-x)
Supplement: Supplementary file 1 — Additional file 1: Table S1. Demographics and clinical manifestations of ARF upon ICU admission in 121 patients with Svv. Table S2. Diagnostic workup upon ICU admission of 121 patients with ARF associated with Svv. Table S3. One-year survival. Table S4. Causes of acute respiratory failure according to the etiology of Svv (n=121). [file 13613_2021_946_MOESM1_ESM.docx]

**Supplementary material**

**e-Table 1**. **Demographics and clinical manifestations of ARF upon ICU admission in121 patients with Svv**

|  | **ALL**  **n=121** | | **Immune ARF**  **n=51** | | **Non immune ARF**  **n=46** | | **Mixed ARF***  **n=24** | | **P** |
| --- | --- | --- | --- | --- | --- | --- | --- | --- | --- |
| **Demographics** | | | | | | | | | |
| Age, year | 62 | [50-75] | 57 | [39-70] | 66 | [57-75] | 73 | [62-79] | 0.0001 |
| Male gender, n (%) | 75 | (62) | 28 | (55) | 33 | (72) | 14 | (58) | 0.214 |
| Diabetes, n(%) | 34 | (28) | 9 | (18) | 15 | (33) | 10 | (42) | 0.067 |
| Cardiovascular disease^a^, n (%) | 63 | (52) | 16 | (31) | 31 | (67) | 16 | (67) | 0.001 |
| Respiratory disease^b^, n (%) | 36 | (30) | 11 | (22) | 19 | (41) | 6 | (25) | 0.394 |
| Immunosuppression^c^, n (%) | 55 | (45) | 7 | (14) | 40 | (87) | 8 | (33) | <0.0001 |
| Severe chronic renal failure^d^, n (%) | 23 | (19) | 1 | (2) | 19 | (41) | 3 | (13) | <0.0001 |
| Chronic dialysis, n (%) | 14 | (12) | 0 | (0) | 13 | (28) | 1 | (4) | <0.0001 |
| **Small vessels vasculitis [Svv]** | | | | | | | | | |
| Time from Svv diagnosis to ICU admission for ARF, months | 1.5 | [0-58] | 0 | [0-0] | 84 | [7-144] | 0 | [0-24] | 0.0001 |
| Svv diagnosis in ICU, n (%) | 55 | (45) | 41 | (80) | 0 | (0) | 14 | (58) | <0.0001 |
| BVAS (Birmingham Vasculitis Activity Score) | 15 | [1-21] | 21 | [15-25] | 0 | [0-4] | 20 | [16-24] | 0.0001 |
| FFS (Five factor score) | 1 | [0-2] | 1 | [0-2] | 0 | [0-1] | 2 | [1-2] | 0.0001 |
| **Clinical presentation upon ICU admission** | | | | | | | | | |
| **Reasons for ICU admission**, n (%) | | | | | | | | | |
| Moderate to severe ARDS  Arterial hypertension | 41  25 | (34)  (21) | 22  7 | (43)  (14) | 9  7 | (20)  (15) | 10  11 | (42)  (46) | 0.033  0.003 |
| Shock | 20 | (17) | 6 | (12) | 13 | (28) | 1 | (4) | 0.018 |
| Neurological (GCS ≤13) | 19 | (16) | 5 | (10) | 11 | (24) | 3 | (13) | 0.144 |
| **Time between first respiratory symptoms and ICU admission, days, n (%)** | | | 4 | [3-4] | 2 | [1-3] | 3 | [1.5-4.5] | 0.001 |
| ≤3 days | 45 | (37) | 11 | (22) | 27 | (59) | 7 | (29) | 0.001 |
| > 3 days | 76 | (63) | 40 | (78) | 19 | (41) | 17 | (71) |  |
| **Extra respiratory symptoms^*^**, n (%) | 104 | (86) | 45 | (88) | 37 | (80) | 22 | (92) | 0.363 |
| **Specific extra respiratory symptoms^#^**, n (%) | 63 | (52) | 38 | (75) | 13 | (28) | 12 | (50) | <0.0001 |
| **Severity criteria on ICU admission** |  |  |  |  |  |  |  |  |  |
| SAPS II | 39 | [27-52] | 37 | [21-51] | 47 | [30-58] | 33 | [26-45] | 0.012 |
| SOFA | 6 | [4-8] | 6 | [3-8] | 6 | [4-9] | 6 | [3.5-6.5] | 0.715 |
| Mechanical ventilation during the first 48hrs, n (%) | 78 | (64) | 33 | (65) | 29 | (63) | 16 | (67) | 0.955 |
| Shock (vasopressors during the first 48hrs), n (%) | 34 | (28) | 13 | (25) | 16 | (35) | 5 | (21) | 0.403 |
| Renal Replacement therapy during the first 48hrs, n (%) | 48 | (40) | 20 | (39) | 17 | (37) | 11 | (46) | 0.768 |

Only the first admission was considered for the analyzis.

^*^ Upon expert review of their charts, 16 of these 24 patients were considered as having a predominant immune cause to ARF, and 8 to have a predominant non-immune cause.

.cardiac or ischemic heart diseaseg and/or renal replacement therapy;

**e-Table 2**. **Diagnostic workup upon ICU admission of 121 patients with ARF associated with Svv**

|  | **N**  **performed** | **All patients**  **N=121** | | **Immune ARF**  **N=67** | | **Non immune ARF**  **N=54** | | **p** |
| --- | --- | --- | --- | --- | --- | --- | --- | --- |
| **Fiberoptic bronchoscopy, n (%)** | **83** | **83** | **(69)** | **53** | **(79)** | **30** | **(56)** | **0.006** |
| **Broncho-alveolar lavage fluid, n (%)*** | 69 | 69 | **(57)** | 47 | (70) | 22 | (42) | 0.002 |
| Hemorrhagic, n [%] | 61 | 38 | (62) | 33 | (79) | 5 | (26) | <0.0001 |
| Total cell count, 10^3/mL | 30 | 248 | [120-480] | 450 | [200-900] | 165 | [80-342] | 0.015 |
| **Chest imaging, n (%)** | **95** | **95** | **(79)** | **60** | **(90)** | **35** | **(65)** | **0.004** |
| Ground glass opacities (CT-scan)* | 95 | 70 | (74) | 53 | (88) | 17 | (49) | <0.0001 |
| Extensive opacities | 121 | 77 | (64) | 53 | (79) | 24 | (44) | <0.0001 |
| Nodules or micronodules | 121 | 26 | (22) | 14 | (21) | 12 | (22) | 0.38 |
| Fibrosis** | 121 | 12 | (10) | 6 | (9) | 6 | (12) | 0.64 |
| Mediastinal lymphadenopathy | 121 | 10 | (8) | 5 | (7) | 5 | (9) | 0.72 |
| Pleural effusion | 121 | 28 | (23) | 17 | (25) | 11 | (20) | 0.75 |
| Pulmonary embolism | 121 | 8 | (7) | 3 | (4) | 5 | (9) | 0.29 |
| **Echocardiography, n (%)** | **121** | **109** | **(90)** | **64** | **(96)** | **45** | **(83)** | **0.026** |
| Cardiac dysfunction*† | 109 | 88 | (81) | 55 | (86) | 33 | (73) | 0.10 |
| **Auto-immunity tests performed, n (%)** | **108** | **108** | **(89)** | **67** | **(100)** | **41** | **(76)** | **<0.0001** |
| Positive ANCA and/or anti-GBM* | 108 | 75 | (69) | 57 | (85) | 18 | (44) | <0.0001 |

Only the first admission was considered for the analysis of the diagnostic workup of immune compared to non-immune ARF.

* Values and % relative to samples or imaging obtained

** Fibrosis [lung distortion, bronchiectasis, honeycombing]

† Systolic or diastolic cardiac dysfunction

**e-Table 3.** **One-year survival**

|  | Univariable | | | Multivariate | | |
| --- | --- | --- | --- | --- | --- | --- |
| Variable | HR | 95CI | *P* | aHR | 95CI | *P* |
| Non-immune ARF | 2.20 | 1.08-4.51 | 0.031 | 2.04 | 0.99-4.18 | 0.052 |
| Time from Svv to ARF, months | 1.0017 | 0.999-1.004 | 0.203 |  |  |  |
| Immunosuppression | 1.90 | 0.94-3.86 | 0.074 |  |  |  |
| Severe renal failure | 2.06 | 0.95-4.46 | 0.066 |  |  |  |
| SapsII ≥ 40 | 2.63 | 1.24-5.56 | 0.011 | 2.47 | 1.17-5.24 | 0.018 |

Only the first admission was considered for the analyzis. After adjustment for time since onset of disease, immunosuppression, severe renal failure and the severity of acute illness score SAPS II (which includes age) in a Cox model, non-immune ARF was marginally associated with a poorer 1-year survival.

The same analyses have been performed using only patients with pure immune or non-immune etiology of ARF. In the Cox model for survival, the etiology of ARF was associated with survival (HR 2.28, Std. Err. .88, z 2.12, P>|z| 0.034, [95% Conf. Interval] 1.06 - 4.86). However, when adjusted for the SAPS2 score (as a dichotomous variable, </>40), the duration (months) of Svv follow-up until ARF, immunosuppression, and severe chronic kidney disease, only the SAPS2 score remained independently associated with survival (HR 3.12, Std. Err 1.36, z 2.61, P>|z| 0.009, [95% Conf. Interval] 1.33-7.35), while the non-immune etiology was marginally associated with survival (HR 1.91, Std. Err .75, z 1.65, P>|z| 0.098, [95% Conf. Interval] .89-4.12).

**e-Table 4. Causes of acute respiratory failure according to the etiology of Svv (n=121)**

| **Immune ARF*** | **ALL**  **N=67** | | | **GPA**  **N=28** | | | **MPA**  **N=24** | | | **EGPA**  **N=9** | | | **GBM**  **N=6** | | | **Ns ANCA**  **N=0** | | **P** |
| --- | --- | --- | --- | --- | --- | --- | --- | --- | --- | --- | --- | --- | --- | --- | --- | --- | --- | --- |
| **Diffuse Alveolar Haemorrhage (DAH)** | **47** | | **(70)** | **21** | **(75)** | | **1921** | **(79)** | | **1(75)** | **(11)** | | **619** | | **(100)** | **(79)** | | **<0.00011** |
| *DAH with pulmonary renal syndrome* | *38* | | *(57)* | *17* | *(61)* | | *1517* | *(63)* | | *1(61)* | *(11)* | | *515* | | **(83)** | *(63)* | | ***0.021*** |
| **Pulmonary or tracheal/bronchial granulomatosis** | **9** | | **(13)** | **9** | **(32)** | | **19** | **(4)** | | **0(32)** | **(0)** | | **00** | | **(0)** | **(3)** | | **0.0030** |
| **Interstitial lung disease** | **11** | | **(16)** | **0** | **(0)** | | **40** | **(17)** | | **7-** | **(78)** | | **04** | | **(0)** | **(17)** | | **<0.00017** |
| **Asthma** | **8** | | **(12)** | **0** | **(0)** | | **00** | **(0)** | | **8-** | **(89)** | | **00** | | **(0)** |  | | **<0.00018** |
| **Myocarditis** | **4** | | **(6)** | **0** | **(0)** | | **30** | **(12)** | | **1-** | **(11)** | | **03** | | **(0)** | **(12)** | | **0.1621** |
| **Non-immune cause associated with immune ARF**** | **16** | | **(24)** | **7** | **(25)** | | **57** | **(21)** | | **2(25)** | **(22)** | | **25** | | **(33)** | **(21)** | | **0.9302** |
| **Non-immune ARF***** | **ALL**  **N=54** | | | **GPA**  **N=24** | | | **MPA**  **N=13** | | | **EGPA**  **N=10** | | | **GBM**  **N=5** | | | **Ns ANCA**  **N=2** | | **P** |
| **Pulmonary Infection (confirmed or suspected)** | **35** | **(65)** | | **15** | | **(63)** | **10** | | **(77)** | **7** | | **(70)** | **1** | **(20)** | | **2** | **(100)** | **0.165** |
| Confirmed | 24 | (44) | | 9 | | (38) | 9 | | (69) | 3 | | (30) | 1 | (20) | | 2 | (100) | 0.08 |
| *Bacterial* | *15* | (12) | | 8 | | (33) | 4 | | (31) | 3 | | (30) | 0 | (0) | | 0 | (0) |  |
| *Viral* | *7* | (6) | | 2 | | (8) | 2 | | (15) | 2 | | (20) | 1 | (20) | | 0 | (0) |  |
| *Pneumocysitis jirovecii* | *5* | (4) | | 2 | | (8) | 3 | | (23) | 0 | | (0) | 0 | (0) | | 0 | (0) |  |
| Suspected | 11 | (20) | | 6 | | (25) | 1 | | (8) | 4 | | (40) | 0 | (0)- | | 0 | (0) | 0.22 |
| **Acute Pulmonary Edema** | **18** | **(33)** | | **8** | | **(33)** | **3** | | **(23)** | **1** | | **(10)** | **5** | **(100)** | | **1** | **(50)** | **0.01** |
| *Left ventricle systolic or diastolic dysfunction* | *12* | (22) | | *6* | | *(25)* | *2* | | *(15)* | *1* | | *(10)* | *2* | *(40)* | | *1* | *(50)* | - |
| *Fluid overload (no cardiac dysfunction)* | *10* | (19) | | *3* | | *(13)* | *2* | | *(15)* | *0* | |  | *5* | *(100)* | | *0* | *(0)* | - |
| **Pulmonary embolism** | **6** | **(11)** | | **3** | | **(13)** | **1** | | **(8)** | **2** | | **(20)** | **0** | **(0)** | | **0** | **(0)** | **-** |
| **Pneumothorax (spontaneous or iatrogenic)** | **4** | **(7)** | | **2** | | **(8)** | **1** | | **(8)** | **1** | | **(10)** | **0** | **(0)** | | **0** | **(0)** | **0.95** |
| **Tumoral^¤^** | **3** | **(3)** | | **3** | | **(13)** | **0** | | **(0)** | **0** | | **(0)** | **0** | **(0)** | | **0** | **(0)** | **0.62** |
| **Immune cause associated with non-immune ARF****** | **8** | **(15)** | | **1** | | **(4)** | **4** | | **(31)** | **0** | | **(0)** | **3** | **(60)** | | **0** | **(0)** | **0.005** |

Only the first admission was considered for the analyzis.

**Immune ARF (N=67)**

*Thirty (45%) of these 67 patients had two or more causes of ARF recorded, including the 16 patients with non-immune cause associated with a predominant immune cause of ARF. ******Non-immune causes were associated with immune ARF in 16 patients, including acute pulmonary edema (n=9), pulmonary infection with microbiological documentation (n=6), and pulmonary embolism (n=2).

**Non-immune ARF (N=54)**

***Twenty-two (41%) of these 54 patients had two or more causes of ARF recorded, including the 8 patients with immune cause associated with a predominant non-immune cause of ARF. ****Immune causes associated with a predominant non-immune cause included alveolar hemorrhage (n=3), pulmonary/bronchial granulomatosis (n=2), interstitial lung disease (n=2), and 1 upper airways obstruction.
